# A Training Smartphone Application for the Simulation of Outdoor Blind Pedestrian Navigation: Usability, UX Evaluation, Sentiment Analysis

**DOI:** 10.3390/s23010367

**Published:** 2022-12-29

**Authors:** Paraskevi Theodorou, Kleomenis Tsiligkos, Apostolos Meliones, Costas Filios

**Affiliations:** Department of Digital Systems, University of Piraeus, 185 34 Piraeus, Greece

**Keywords:** mobile sensor-based applications, usability, user experience, sentiment analysis

## Abstract

Training blind and visually impaired individuals is an important but often neglected aspect of Assistive Technology solutions (ATs) that can benefit from systems utilizing multiple sensors and hardware devices. Training serves a dual purpose as it not only enables the target group to effectively utilize the ATs but, also, helps in improving their low acceptance rate. In this paper, we present the design, implementation, and validation of a smartphone-based training application. It is a form of immersive system that enables users to learn the features of an outdoor blind pedestrian navigation application and, simultaneously, to help them develop long-term Orientation and Mobility (O&M) skills. The system consists of an Android application leveraging, as data sources, an external high-accuracy GPS sensor for real-time pedestrian mobility tracking, a second custom-made device attached to traffic lights for identifying their status, and an ultra-sonic sensor for detecting near-field obstacles on the navigation path of the users. The training version running as an Android application employs route simulation with audio and haptic feedback, is functionally equivalent to the main application, and was used in the context of specially designed user-centered training sessions. A Usability and User Experience (UX) evaluation revealed the positive attitude of the users towards the training version as well as their satisfaction with the skills acquired during their training sessions (SUS = 69.1, UEQ+ = 1.53). Further confirming the positive attitude was the conduct of a Recursive Neural Network (RNN)-based sentiment analysis on user responses with a score of 3 on a scale from 0 to 4. Finally, we conclude with the lessons learned and the proposal of general design guidelines concerning the observed lack of accessibility and non-universal interfaces.

## 1. Introduction

The goal of any training procedure targeting the navigation of blind and visually impaired individuals is to make them as independent as possible. However, it should not only be focused on learning to use a specific AT solution but also on enabling the target group to acquire Orientation and Mobility (O&M) skills. The proliferation of smart devices integrated with a number of sensors in combination with robust text-to-speech functionalities and multisensory haptic and audio-based interfaces allows users to achieve a threefold goal: (1) to make them independent while navigating in familiar and unfamiliar outdoor spaces; (2) to develop training tools that ease and streamline the process of learning to use AT solutions; and (3) to acquire useful long-term O&M skills. Another underappreciated factor of training is the possibility of contributing to the improvement of the AT solutions’ adoption rate [1].

It is critical for people with blindness to develop cognitive and mental map building skills. Specifically, the research literature on blind people’s Orientation and Mobility (O&M) covering both familiar and unfamiliar places [2] describes the requirement of providing the information on a perceptual and conceptual level, which are an integral part of any O&M training course. For the former, since the information received from the visual channel is inadequate, the individuals need to leverage the rest of the functioning senses including the touch, auditory, and olfactory channels to perceive the dangers of the environment. For the latter, blind people acquire and develop suitable cognitive strategies and skills based on their abilities and preferences [3].

Common approaches to facilitate the development of orientation skills involve the use of conventional tools such as the cane and other navigational approaches including preplanning and in-situ aids. The category consisting of the preplanning aids uses tactile maps, verbal descriptions, physical models, digital audio, and tactile screens as mediums of communicating information about the environment before the users’ arrival [4,5,6] while the category of in-situ aids provides the information about the environment as users are present in it. The latter employs obstacle detection [7,8,9,10] and tactile vision substitution systems [11], embedded sensors in the environment [12,13], and navigation systems [14,15]. 

However, the usage of these two categories of aids has various drawbacks. Tactile and interactive maps and models, despite their demonstrated effectiveness in the research [16,17,18,19,20], are restricted in the sense of requiring larger devices and/or tactile overlays [18,21,22], have low geographical information resolution, are also difficult to manufacture and update their spatial information, and they are scarce. Furthermore, blind people are less likely to employ preplanning aids in everyday life because of these constraints. 

On the other hand, the usage of in-situ aids is associated with an increased risk as most of them are based on auditory feedback which could potentially affect the users’ attention when navigating in real spaces. To circumvent the difficulties, one viable option is to obtain spatial and route knowledge indirectly before navigation [16,17]. Earlier research has demonstrated the power of virtual navigation [23,24] in virtual environments. The core idea is to allow the blind individual to experience unfamiliar regions through virtual walking while remaining in a safe, regulated setting. Since the complexity of this virtual environment can be dynamically modified, it can be used to provide training scenarios of varying complexity ranging from simple to realistic settings [21]. A common approach for the latter [22,25] is to employ 3D audio navigation via egocentric exploration. 

Various works have shown the effectiveness of the virtual environments approach. The work presented in [26] was the first to highlight the success of integrating a virtual environment application into O&M training sessions for improving the O&M skills of individuals with blindness and visual impairments. She demonstrated an improvement in the target group in performing orientation tasks in real space. Furthermore, the strengths of the tools that utilize virtual environments are threefold: a training simulator for O&M; a diagnostic tool for O&M specialists to track participants’ spatial behavior; and a technique for advanced exploration of unfamiliar spaces. 

Later, Guerreiro [27], studied the effect of using virtual navigation on building route knowledge and to what extent the acquired knowledge can be transferred to the real world. They found that during virtual navigation users were able to accelerate the learning process of short routes and gradually improve their knowledge of both short and long routes. Afterwards, the users were able to transfer the acquired knowledge from virtual navigation to the real world and successfully complete unassisted navigation tasks. 

In [28], extending her work, the researcher investigated how virtual environments affect individuals who are blind and visually impaired in exploring, creating cognitive maps, and carrying out activities requiring spatial orientation in real situations. The findings of the study demonstrated that multisensorial VR systems impact the same or even better spatial abilities of the individual when compared with exploring space in the real world. However, it does take some time before the user is able to quickly transfer spatial knowledge from the virtual environment to the real world. These findings emphasize the need for such an orienting tool, particularly when it is impossible to independently explore a new environment. Analogous outcomes were discovered in other VR orientation system studies, [26,27,29,30,31,32,33,34,35,36,37,38,39]. 

Despite the multiple benefits of employing VR-based solutions, they are not without limitations. The main disadvantage of this approach is its high cost [25,26] regarding their complexity in developing and in the required tooling and equipment which makes it prohibitive in low-income areas.

To alleviate the problems of both conventional and VR-based approaches, and based on the results of [40] demonstrating the effectiveness of smartphone-based approaches in improving navigational skills, route learning, and public transit, we tried to explore simpler and more cost-effective solutions based on smartphone devices without making any compromises regarding the quality and effectiveness of the provided learning process. Our attempt resulted in an Android-based application functionally equivalent to our main application for outdoor navigation [41] where simulates a navigational route that the current user or previous ones have traversed in the past. The proposed solution has the benefit of being easily demonstrated to both instructors and trainees, does not require the availability of special infrastructure, and is cost-effective as low-end Android devices can support the application’s operation. The conduct of a Usability and UX evaluation confirmed the above statements and demonstrated, also, that the users consider the training application to be useful towards learning to operate the main application’s features as well as easy to use, efficient, and dependable. This was further validated by a Recursive Neural Network sentiment analysis algorithm on users’ responses.

In this paper, we present the design, implementation, and validation of a mobile-based training application enabling blind users to learn the features of the main outdoor pedestrian navigation application and develop O&M skills. Section 2.1 presents the user-centered process employed in the design phase of both the main and training application as they are currently implemented. Next, the technical description of the main application (Section 2.2) is presented followed by the relevant description concerning the training version (Section 2.3). Section 2.4 describes the methodology and measures employed for evaluating Usability and User Experience as well as the tools and methods used for sentiment analysis. Section 3 demonstrates the results of the previous section. Namely, it presents the results of Usability as measured in terms of efficiency, effectiveness, and with the help of the widely used SUS questionnaire, the results of UX as measured with the help of the popular modular UEQ+ questionnaire and the results of sentiment analysis as produced by the Stanford CoreNLP Natural Language Processing Toolkit framework. Section 4 presents a discussion of guidelines/lessons learned. Finally, Section 5 concludes the paper.

## 2. Materials and Methods

### 2.1. Design Process

For both the main application and its companion training version, we applied a cognitively informed design process [42]. It differs from the common engineering approach as it further considers the cognitive procedures utilized in systematic problem solving as part of the design process as well. Moreover, it emphasizes the participation of the immediate beneficiaries in the design process, and it employs safety, reliability, reinforcement, and preferences as guiding principles. 

Crucial to the success of the design is to understand the needs of the blind and visually impaired in the broader socioeconomic context and the constraints it imposes [43]. With the aid of interviews, we achieved that and identified the various cognitive processes (e.g., allocentric, egocentric, and the like) that are employed during navigation. Furthermore, we recognized various psychological constructs (interest, focus, enjoyment, and the like) that can be used to create more appealing and acceptable applications [44,45,46,47,48].

### 2.2. System Description

The proposed system allows for safe and highly precise outdoor blind pedestrian navigation without requiring the mandatory use of tactile ground surface indicators. The system employs voice instructions to continuously inform the user about the status and progress of the navigation and the various obstacles found along the navigational path. Central to this system is an Android application that aggregates data from two different sources, namely an external high-precision GPS receiver tracking real-time pedestrian mobility, a second custom-made external device consisting of an ultrasonic sensor and a servo mechanism that resembles a sonar device in its functionality, and a third custom-made waterproof device attached to traffic lights for identifying their status. The external high-precision GPS receiver (NEO-M8N) leverages information from multiple (up to 16) satellites enabling the system to provide centimeter-level location precision (~10 cm). From trials reported in [41], besides demonstrating its excellent precision unprecedented for blind pedestrian navigation, it also demonstrated significant improvements in the observed deviation from the actual user location when compared to the smartphone-integrated GPS receiver. The deviation is a crucial factor that can negatively impact the robustness of pedestrian navigation. Specifically, the deviation of the external receiver is measured to be less than 0.4 m, when receiving signal from 11 satellites, while the readings from the smartphone-integrated GPS receiver deviate in the order of 10 m. By utilizing this receiver, the application has information on the user’s latitude, altitude, speed, bearing, date, time, and number of satellites used. 

The ultrasonic sensor integrated to the obstacle detection subsystem is the widely used in robotic applications HC-SR04. It works optimally between 2–400 cm within a 30-degree cone and is accurate to the nearest 3 mm. Its selection was based on its cost-effectiveness, small weight and size besides its functional characteristics. In order to transmit the ultrasonic burst, a single control pin has to be set high for 10 us. As soon as that happens, the output data pin, responsible for taking the distance measurements, is set to high and remains in that state until the transmitted ultrasonic burst is detected back. In [49] the employed sensor demonstrated a robust performance in sparse outdoor city environments with wide pavements while in densely populated city environments other sensors with narrow beam widths performed significantly better. This is due to the fact that the important factor for achieving reliable results is the width of the beam and not the detection distance.

The third external device is used to detect the change of the traffic light status and communicate the event to the main Android application. The technical details of this device will not be presented as they are part of a pending patent. Traffic light status changes can be detected with no latency and sent via Bluetooth to the main Android application. There, a loop responsible for processing those events and turning them into actionable instructions runs continuously. The processing phase until issuing the instruction takes around 30 ms. Overall, it takes 100 ms for the system to receive the traffic light status change and calculate the vector representing the user’s movement used in the navigation process. 

The user interacts with the system via an appropriately designed voice interface to enable fast and accurate interaction. Upon the user’s selection of a destination, the system requests information from the Google Maps service and feeds the received data into a novel routing algorithm [48]. In case the user selects to include the use of public transportation, the application requests from the Athens Public Bus Transportation (OASA) real-time telematics service information relevant to the available schedules and bus stops. The algorithm processes the totality of the received data to plan a high-precision navigation route that may or may not include public transportation and issues a high-level description of the overall route. At the same time, the external high-precision GPS receiver continuously transmits via Bluetooth the coordinates to the Android application where two processes allow the system to adapt to the dynamically evolving environment. The first one is responsible for navigating the user via the use of the aforementioned Google Maps service and for updating the list of the available public transportation. The second process is responsible for utilizing the data received from the GPS and the sonar sensor for reporting user position with a negligible margin of error (<1 m) and for obstacle detection, respectively.

**Figure 1:**
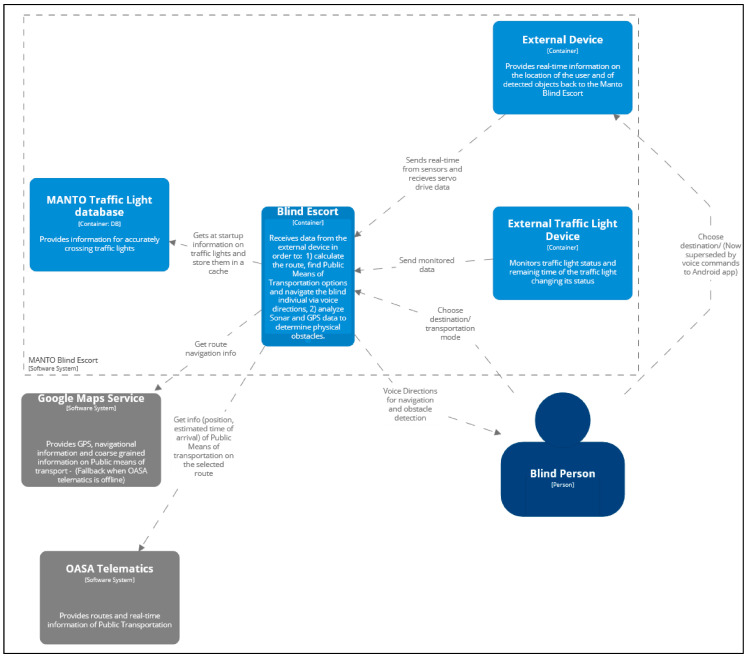
The application’s architectural diagram.

The application transmits voice instructions in order to simultaneously ensure the correct and safe navigation of the users and to give feedback about potential obstacles on the navigational route. The instructions, information, and options requesting user response emitted via the application are better experienced through the use of bone-conducting headphones. In this way, sounds from the enclosing environment are not suppressed, enabling users to remain aware of the dangers which are critical for their safety. Figure 1 depicts the architecture of the proposed system at a high level. The operation of the reliable ultrasonic obstacle recognition subsystem is presented in detail in [49].

### 2.3. Training Application System Description

#### 2.3.1. General Description

For people who are blind and visually impaired, independent navigation is difficult, especially in unknown environments. Navigation assistive technologies attempt to provide further assistance by directing users or raising their awareness of their surroundings, but precise solutions are still few. Even when adequate solutions exist, usually there is no companion application that would make it easier for the users to understand the supported features of the proposed solutions. As a response to this inadequacy, we developed for our application an interactive virtual navigation software solution that supports both Android smartphones and PCs. The latter is a user-friendly bundle of the Android emulator that allows users to utilize the same application on a PC as well. The end goal is to ease the engagement with assistive technologies and increase the effectiveness of their usage since spatial information can be obtained indirectly (before navigation).

In the beginning, the simulation environment was just a debugging tool, but its value as a functional educational tool soon became apparent and, thus, evolved into a full-fledged training environment. This mode of operation avoids the hazards of trials in real scenarios and, as a result, it assures safety and trust, both of which are requirements of fundamental importance. The main functionality of the simulation application is the provision of the capability to replay/rehearse a navigation route without having to move along that route. Since the training version’s overall functionality is equivalent to that of the main application, it allows everyone to practice at their own pace, with an increased level of comfort and devoid of any external limitation (instructor, escort). The simulation application also provides the capability to become acquainted with the obstacle detection mechanism found in the main application. Virtual obstacles can be placed in the navigational path of the simulated route where special audio pitches or haptic-based feedback is used to indicate their presence. The intensity of the pitch or of the vibration increases or decreases depending on whether the user is approaching or moving away from a face-fronting obstacle. When both a navigational instruction and an obstacle detection pitch need to be emitted, the navigational instruction takes precedence unless the obstacle’s distance is below a defined threshold value. Overall, the simulation tool is a form of an immersive system [50].

The simulation tool utilizes a custom-made JSON structure that describes locations found on the replayed routes. During the simulation, the application will read the points the user has passed one by one and will issue directions both for navigation reasons and for informing the user about surrounding Points of Interest (POIs) as if the user were physically walking through them. 

The instructors will need to build the JSON file with this structure each time a new path is added either manually or by traversing the route themselves. In our example, five points have been placed for illustrative purposes. The order must be respected as the points are traversed sequentially. In addition, we assume that the user starts from the first point in the list.

The JSON file follows. As can be seen, rootingPaths is an array of UserPoints with the following format:
“rootingPaths”: [ {  “id”: 1,  “lat”: 19.427874,   “lng”: 25.464897 }, {  “id”: 2,  “lat”: 19.427875,   “lng”: 25.464899 }, {  “id”: 3,  “lat”: 19.427876,   “lng”: 25.464895 }, {  “id”: 4,  “lat”: 19.427877,   “lng”: 25.464894 }, {  “id”: 5,  “lat”: 19.427878,   “lng”: 25.464893 }],“destination”: { “lat”: 19.427890, “lng”: 25.464890}

Each UserPoint is a JSON object that contains three fields as seen below.
{ “id”: 5, “lat”: 19.427878, “lng”: 25.464893}

The fields lat and lng are the geographic coordinates of a point that is part of the route while the id field uniquely identifies a point in the array of UserPoints. The final destination of the route has the following format:
“destination”: { “lat”: 19.427890, “lng”: 25.464890}

#### 2.3.2. Graphical User Interface (GUI) during Training Navigation

**Figure 2:**
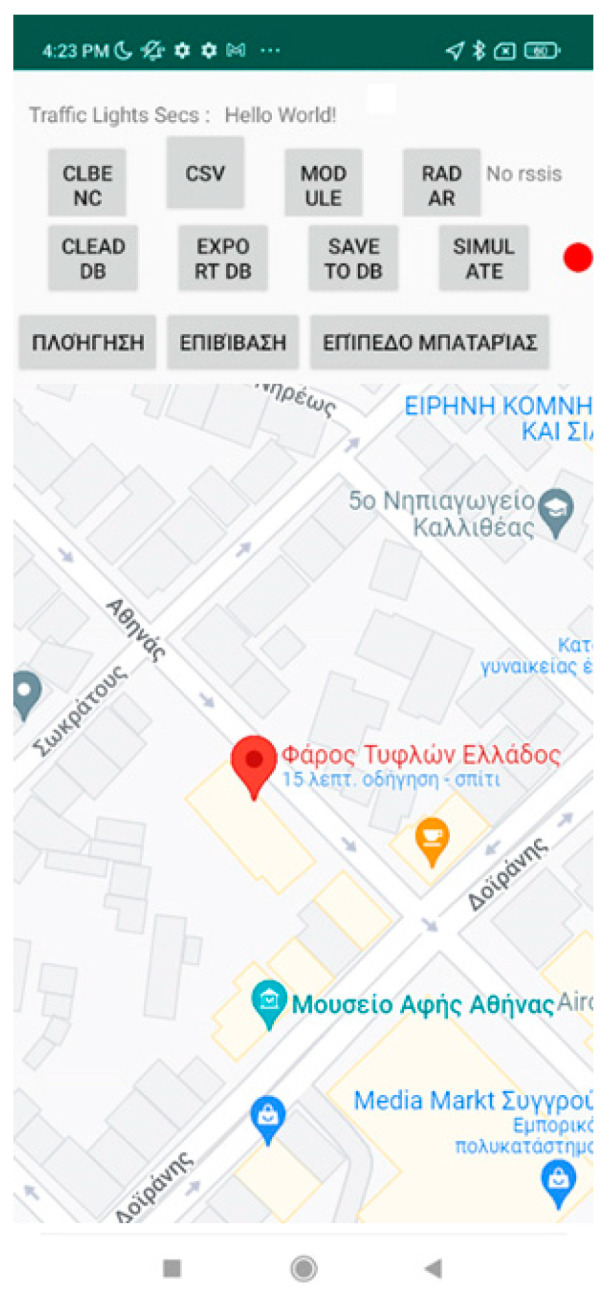
Application’s Training version—Main screen. It consists of three parts: the upper part contains the training app’s menu and the rest depicts the map, the user’s location and the navigational route. The Greek text on the last three buttons says, from left to right, “Navigation”, “Boarding”, and “Battery Level”.

**Figure 3:**
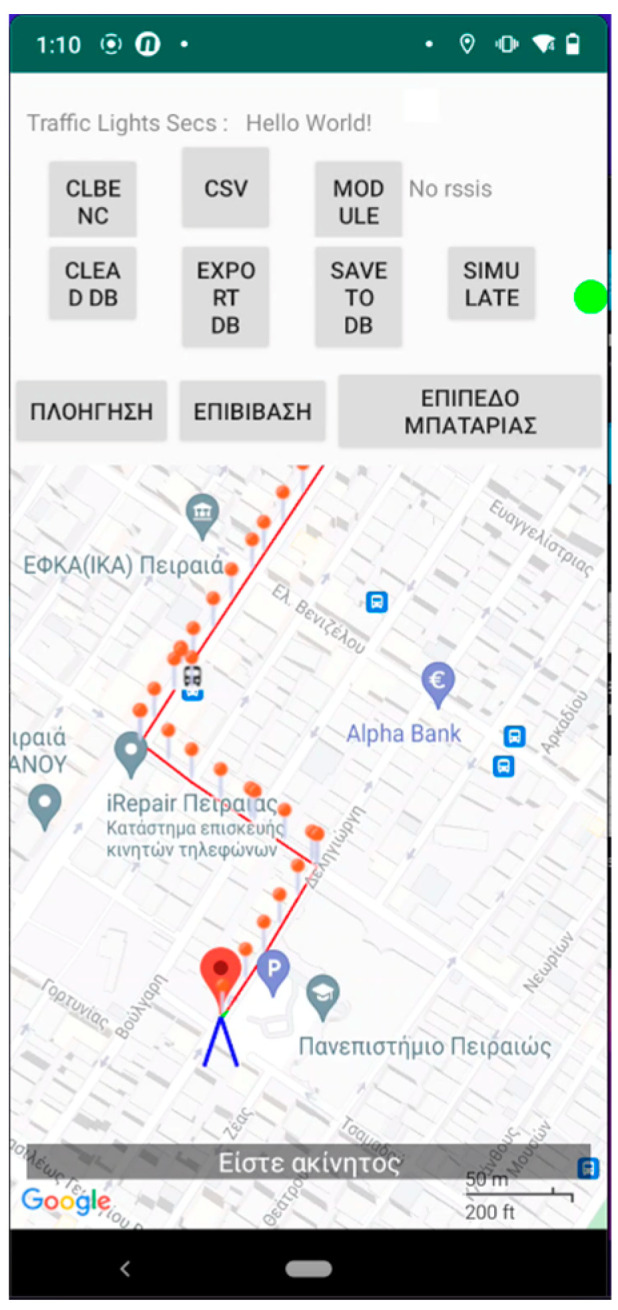
Application’s Training version—Route selected. The entire simulated route is depicted with dotted pins. These serve as preselected locations that the instructor can set as starting points for navigation. The message at the bottom of the image says in Greek: “You are stationary” as the replay of the route has just begun.

Figure 2 and Figure 3 present an excerpt of the GUI elements of the training version as a scenario is being replayed. It is almost identical to the main application except for the upper part of the screen as shown in Figure 2 and the pins (dots) depicted on the map representation (Figure 3) of the route. 

The menu on the upper part (see either Figure 2 and Figure 3) allows the instructor to access the features of the app. Specifically, it provides the route simulation features such as (1) selecting the JSON file to load a route via the “Navigation” button located on the first position from the left of the bottom three (described in Greek), (2) the button responsible for simulating the process of boarding a bus and the battery level notification (second and third from left of the bottom three, respectively), (3) the feature to record, import, and export routes made by the blind and visually impaired users, and, finally, (4) starting and stopping the simulation of the selected route. 

#### 2.3.3. Simulated Route Navigation

**Figure 4:**
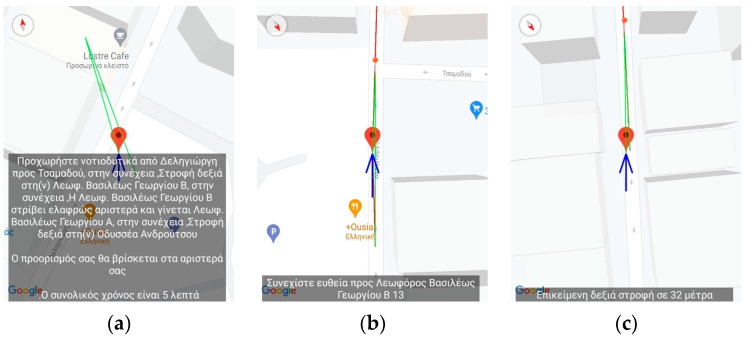
Application’s Training version—Starting the virtual navigation. The figures display the navigation messages issued from the application. The translation of the text is as follows: (**a**) Summary of the entire route, (**b**) “Keep moving straight ahead towards Vasileos Georgiou B 13 Avenue, (**c**) “Upcoming right turn in 32 m”.

**Figure 5:**
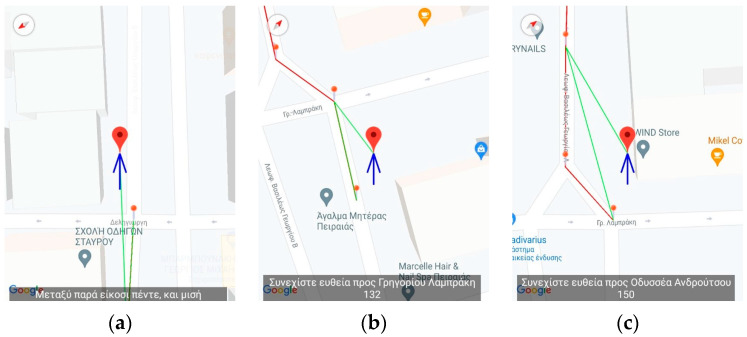
Application’s Training version—Midway through the virtual navigation. The figures display the navigation messages issued from the application. The translation of the text is as follows: (**a**) The user deviates from the correct navigational pat and the application issues the following error-correction message: “The correct direction is between 6 and 7 o’clock”, (**b**) “Continue straight ahead towards Grigoriou Lampraki 132”, (**c**) “Continue straight ahead towards Androutsou 150”.

**Figure 6:**
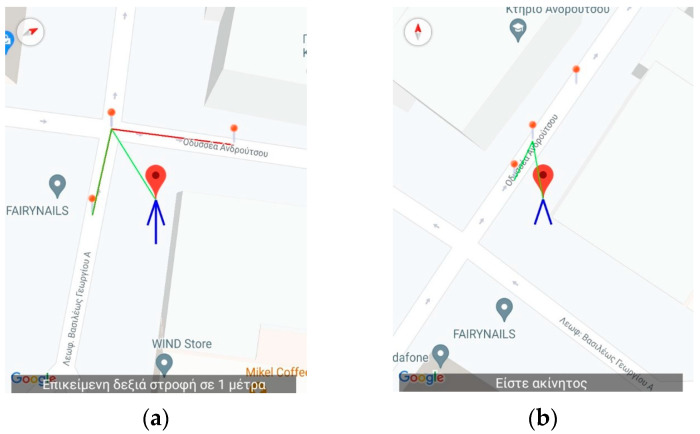
Application’s Training version—End of virtual navigation. The figures display the navigation messages issued from the application. The translation of the text is as follows: (**a**) Turn right in 1 m”, (**b**) “You have reached your destination”.

Figure 2, Figure 3, Figure 4, Figure 5 and Figure 6 present the output of the application when either the instructor or the user has selected to simulate a route already recorded. When the route has completed loading, the application inserts, at regular intervals, pins which the instructor can select to reset the simulated navigation. As the simulated walk progresses, the messages are replayed in the same order they were encountered during the recording phase. 

Figure 4, Figure 5 and Figure 6 present a recorded route from the vicinity of the Piraeus starting from Deligiorgi 114 and ending at Odyssea Androutsou 150. In these figures, the main capabilities of the application are represented as various events unfold during the navigation. The following messages are issued starting with the description from the leftmost depiction in Figure 4:

1. Summary Description of the full navigational route and estimation of the time of arrival at the destination (Figure 4a).
2. “Keep moving straight ahead towards Vasileos Georgiou B 13 Avenue” (Figure 4b).
3. “Upcoming right turn in 32 m” (Figure 4c).
4. The user, ignoring the voice command to turn right, turns left and the application detects that and provides error-correction information by issuing the following message: “The correct direction is between 6 and 7 o’clock” (Figure 5a).
5. “Continue straight ahead towards Grigoriou Lampraki 132” (Figure 5b).
6. “Continue straight ahead towards Androutsou 150” (Figure 5c).
7. “Turn right in 1 m” (Figure 6a).
8. “You have reached your destination” (Figure 6b).

**Figure 7:**
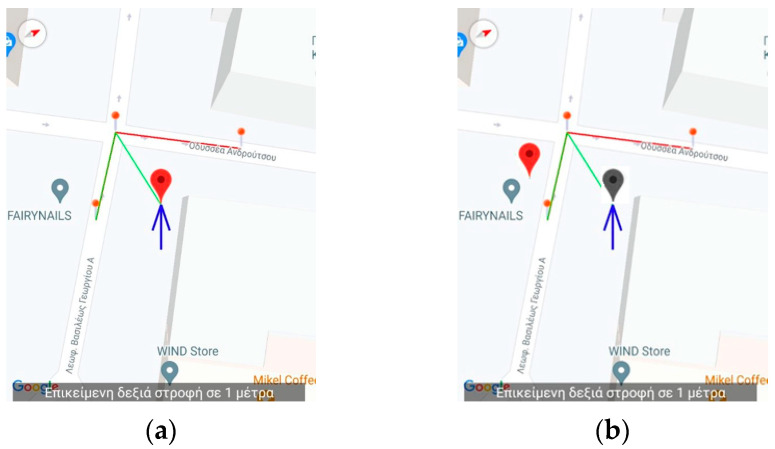
Application’s Training version—Snapshot from a simulated route (**a**) while it is being replayed. The instructor pauses the replay and selects to move the position of the user in an arbitrary location on the map in order to demonstrate the behavior of the app when a wrong turn event occurs. Upon selecting the new position, the old is grayed out (**b**). The text in both figures displays the message when the instructor paused the replay: “Upcoming right turn in 1 m.

In addition, the app gives the capability to the instructor to arbitrarily place the simulated position anywhere on the map for demonstration purposes. Figure 7 depicts the case were the instructor placed the simulated position at a different location than the simulated navigation route suggests. This is performed to demonstrate to the blind user the behavior of the application when a wrong turn is taken. When the new position is inserted, the old is grayed out as shown in Figure 7. The application will respond with a correction message using instructions based on the hands of the clock identical to the one that the main navigation application would give in this case (i.e., the correct direction is between 6 and 7 o’clock).

#### 2.3.4. Passing Near Traffic-Light Crossings

**Figure 8:**
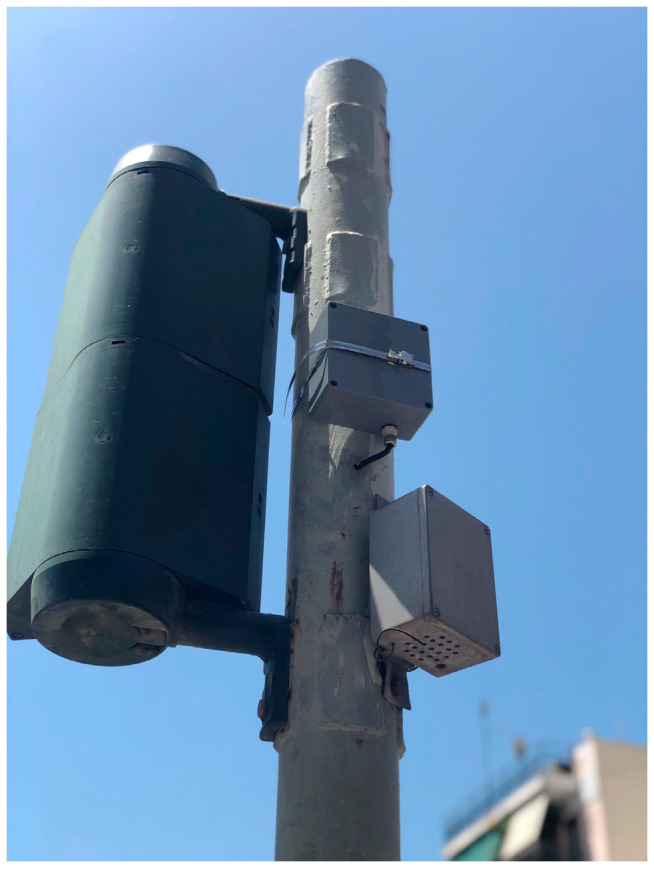
Specially mounted external device on a real traffic light located at the junction of Doiranis and Athinas in Kallithea.

Similar instructional scenarios can be performed for the case of passing near traffic light crossings and for the case of combining the navigational route with public transportation. Figure 8 depicts the traffic light at the junction of Doiranis and Athinas in Kallithea were the second external device designed by the research team is mounted.

**Figure 9:**
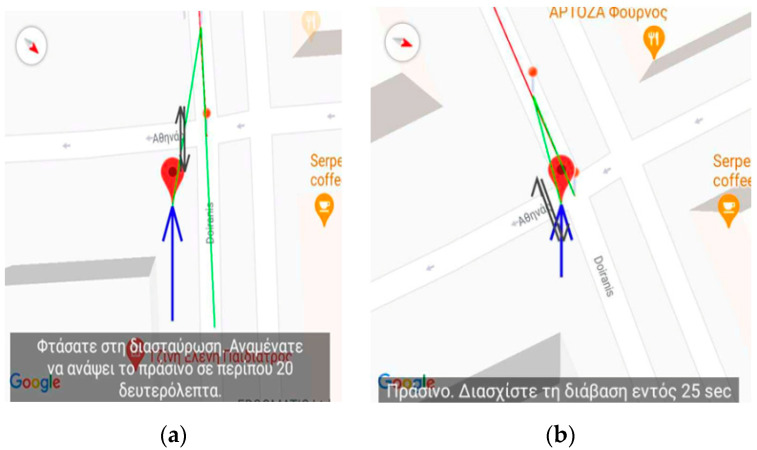
Application’s Training version—Snapshot from simulating the passage of the crossing near the traffic light of the Doiranis and Athinas junction in Kallithea. The translation of the issued message displayed on (**a**) is: “You have reached the intersection. Wait for 20 s until the traffic light status is green” while for (**b**) is: “Green. You can pass the crossing. Remaining time 25 s”.

The application in combination with the latter device helps the blind user to pass traffic light crossings by detecting the traffic light status changes and by reporting the duration in which the traffic light remains in the green status. Figure 9a depicts the user approaching the traffic light and the messages issued. When the user has reached the traffic light, the application, then, informs the user of the event and instructs the user to wait for 20 s, the time required for the traffic light to change to the green status at the time of the recording. When the traffic light is green, then the application issues a message requesting the user to pass the traffic light crossing in 25 s as shown in Figure 9b, which is the time until the green status changes to another state at the time of recording.

#### 2.3.5. Enhanced Route Navigation with Bus Transportation

**Figure 10:**
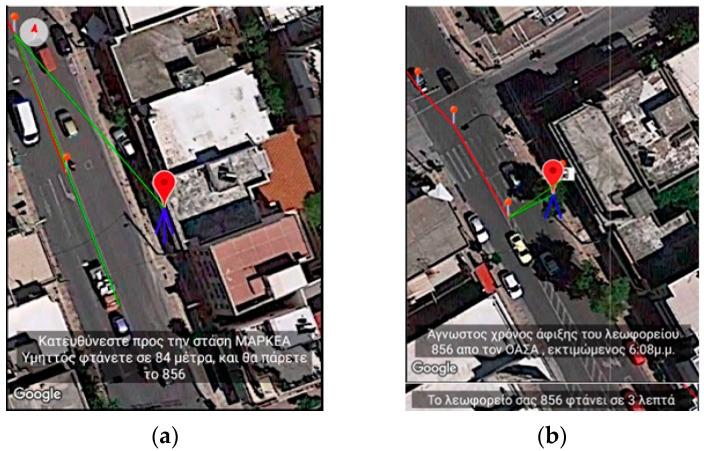
Application’s Training version—Snapshot from simulating pedestrian navigation coupled with public means of transportation. (**a**) depicts the following message: “Heading toward MARKEA—Ymittos stop at 84 m distance. You will take bus line 856” while (**b**) the message: “Unknown time of arrival for the bus line 856 from the Telematics Service”, and “The bus line 856 is estimated to arrive in 3 min”.

**Figure 11:**
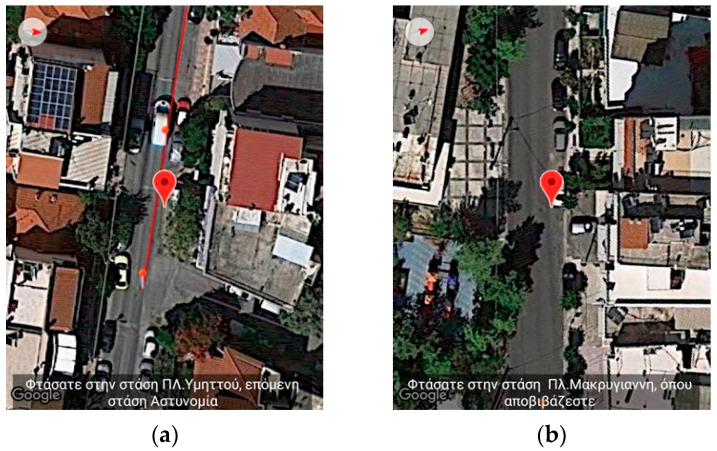
Application’s Training version—Snapshot from simulating pedestrian navigation combined with public transportation. (**a**) depicts the message when reaching and passing intermediate bus stops “You reached Ymittos square stop. Next stop Police” while (**b**) displays the message “You reached Makrigianni square stop. You exit here”.

Figure 10 and Figure 11 depict the case of using a bus as part of pedestrian navigation. In contrast to the other cases where street view is being used, the snapshots are taken with the satellite view. 

The user in this recorded session takes bus line 856 from the MARKEA—Ymittos stop heading to Makrigianni square stop. When the user starts approaching the first bus stop, the application starts informing the user about the distance remaining. It emits the message: “Heading toward MARKEA—Ymittos station. Remaining 84 m. You will take bus line 856” (Figure 10a). While the user is waiting for the bus to arrive, the application informs the user about the estimated time of arrival based on input from the telematics service supporting the bus. In this recording, the messages the user hears are “The bus is estimated to arrive at 6:08,” followed in a few moments by the message “The bus will arrive in 3 min” (Figure 10b). To simulate the button required to indicate the user is on board the bus, the training application has a relevant button as described in a previous section. As the user passes intermediate bus stops, the application informs the user about those events (“You reached Ymittos square stop. Next stop is Astinomia”, Figure 11a). Finally, when the user reaches the destination, the following message is emitted: “You reached Makrigianni square stop. You exit here” (Figure 11b). When the replay of the recorded route completes, the user can restart either from the beginning or from any other point of the route.

### 2.4. Methodology

The training of the blind and visually impaired with the aforementioned educational tool took place on the premises of the BlindHouse of Greece. The training course, which was part of the Orientation and Mobility courses, included a series of lectures and demonstrations that explained Orientation and Mobility (O&M) techniques, how to navigate routes, and where to find information about Public Transportation, or other conveniences and, finally, all of the above in conjunction with the proposed technology. The selection of the O&M class as a venue for our training course was the result of numerous meetings and interviews between the research team and the O&M instructors to get acquainted with the ins and outs of the course as well as the different educational tools that are used. 

The sessions were held once a week for blind and visually impaired users to familiarize themselves with the training version of the application. The sessions were either private or organized as small group classes depending on the needs of the trainees and the limited resources, providing at the same time socialization opportunities. The instructor, a permanent employee of the BlindHouse of Greece who had previously received training from our research team, would start the exhibition of the application by informing the trainees about the existence of two separate versions of the application having almost identical functionality. Particularly, the instructor described both the supported functionality and generic capabilities as well as the common mistakes of users, in order to accelerate the learning procedure. Furthermore, during the sessions, seasoned users, if present, would frequently assist others by providing step-by-step instructions while performing the activities on their own devices and waiting for others to complete each step. Overall, the participants favored an active style of learning over handing up their gadgets to others.

In order to assess the training application, we created a procedure including carefully defined tasks as well as questionnaires. The sample consisted of 25 individuals from the population of the blind and visually impaired including both males and females with varying causes of disabilities, with ages between 30 and 60 years old as well varying digital sophistication skills. The bulk of the participants had little to no digital expertise, which explained the demand for training sessions.

**Figure 12:**
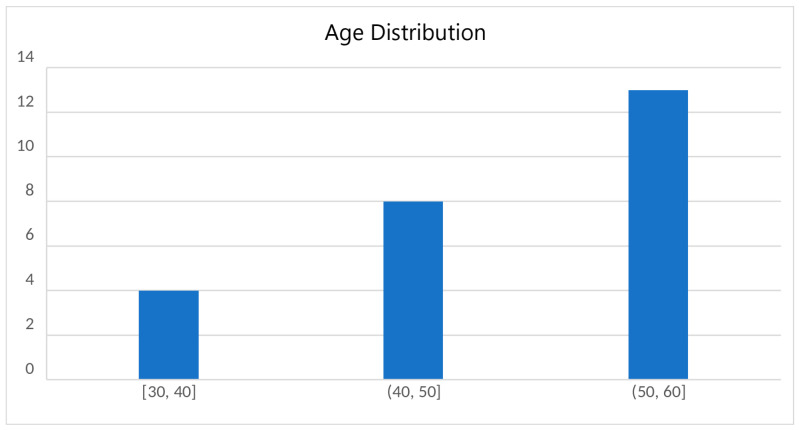
Participants’ age distribution.

Figure 12 presents the distribution of the participants’ ages.

In order to evaluate the Usability and User Experience of the training application, we capitalized on the results from a previous research attempt [48] to search the literature for commonly used tools and methods, and definitions as well. There we discovered that there is neither consensus on the method used to assess both Usability and User Experience nor a commonly agreed definition of the concepts involved as a matter of fact, and typically, a combination of tools and methods are employed, the majority of which are based on questionnaires. For example, UX is a term that many researchers and practitioners use to incorporate different concepts [51]. It can include a range of dynamic concepts, such as traditional usability (e.g., [52,53] as well as affective, emotional (e.g., [54,55,56,57]), hedonic (e.g., [58,59]), experiential (e.g., [51,52,53,54,55,56,57,58,59,60]), and aesthetic dimensions (e.g., [61]. Furthermore, UX, according to ISO 9241-210:2019 [62], includes users’ emotions, beliefs, physical and psychological responses, and it is also the result of system performance, brand image, presentation, the user’s internal and physical state resulting from prior experiences, skills, personality, and attitudes, among others.

Despite the disagreement in the field, a prominent definition that stands out is the ISO/IEC 25,010 2011 [63] standard where usability is defined as “the degree to which a product or system can be used by specified users to achieve specified goals with effectiveness, efficiency and satisfaction in a specified context of use”. This definition of Usability has the added feature of incorporating User Experience as one of its components under the name of satisfaction [48]. In our experiment and in accordance with the above standard, we utilized the measures of effectiveness and efficiency to quantitively assess Usability and the modular extension of the popular User Experience Questionnaire (UEQ+) to assess the dimension of satisfaction. Without restricting the choice of tools from the above definition, we utilized the widely popular System Usability Scale (SUS) to qualitatively assess Usability as well. In our effort to further understand how well received was the training process, we conducted sentiment analysis, a common technique used in assessing product reviews. Finally, independent of the previous efforts, we employed a semi-structured questionnaire where the participants freely gave feedback to the research team concerning the functionality of both the training and the main version of the application. 

The evaluation results from both Usability and UX can be used to make statements about the application’s overall behavior and, to some extent, more general statements for those types of applications despite being deduced from a relatively small sample. Finally, the participants are representative of the population of the BlindHouse of Greece in terms of age, gender, age of visual loss, and capability to utilize digital devices.

#### 2.4.1. Effectiveness and Efficiency

Effectiveness and efficiency measure the degree to which users can complete a task and the time it takes users to complete a task, respectively. For our case, we employed the following tasks [48] where we requested that users (1) select and traverse a route “virtually”, (2) combine a route with the use of Public Transportation, and (3) pass a traffic light crossing. The participants completed these tasks after they had been shown earlier how to utilize the training application. Finally, for the calculation of the above, the following types were used:

(1) $$Effectiveness=\frac{total\;\#\;of\;tasks\;successfully\;completed}{total\;\#\;of\;tasks\;undertaken}=\frac{\sum_{l=1}^{U}\sum_{i=1}^{Μ}task_{li}}{U*M}\;$$

where *U* = *#* of participants, *M* = *#* of tasks per participant and *task_li_* = *i*-th task of the *l*-th user.

(2) $$Efficiency=\frac{\sum_{j=1}^{U}\sum_{i=1}^{M}tasks_{ij}t_{ij\;}}{\sum_{j=1}^{U}\sum_{i=1}^{M}t_{ij\;}}\times 100\%$$

where $t_{ij}$= $EndTime_{ij}-StartTime_{ij}$, which in turn, *EndTime ij* is defined as the time required for the *i-th task of the j-th user* to be completed successfully or the time until the user quits.

#### 2.4.2. UEQ+ Standardized Questionnaire

To the best of our knowledge, there are no questionnaires available that evaluate the user experience of blind and visually impaired individuals. UEQ+ was selected to address the issue of having predefined general-purpose questionnaires without the ability to selectively examine specific aspects of a software artifact. It promotes modularity as it provides a number of scales to select from, each decomposed into four items, evaluated on a Likert scale that ranges from 1 to 7. Furthermore, each scale is evaluated by the participants for its relevance or importance. Alongside the modular questionnaire, the UEQ+ framework provides a statistical tool to ease the analysis. Finally, the set of scales selected for the User Experience evaluation is the following: Efficiency, Perspicuity (educability), Dependability, Adaptability, Usefulness, Trustworthiness of Content, and Response Behavior. For a detailed description of their meaning, the reader can refer to [48].

#### 2.4.3. System Usability Scale (SUS)

The System Usability Scale (SUS) was proposed in 1986 by Brooke as a “quick and dirty” tool to measure usability. Since then, it has become one of the most popular questionnaires used in subjective assessments of the usability of software products [64]. SUS is a ten-item questionnaire evaluated on a 1 to 5 Likert scale and, according to a recent study, it accounts for 43% of post-test questionnaire usage of unpublished studies [65]. Despite the original characterization of “quick and dirty”, in a study of 2324 cases conducted by Bangor, Kortum, and Miller, SUS was found to have an alpha coefficient of 0.91. Furthermore, they provided some evidence of the validity, both in the form of sensitivity and concurrent validity [66]. Finally, Appendix A presents the SUS questionnaire utilized in this study.

#### 2.4.4. Semi-Structured Questions

We have designed a seven-point Likert scale questionnaire to get feedback about the training version’s functionality and to better comprehend its challenges. This format was chosen as it captures the users’ views despite being less amenable to statistical analysis. The reader can learn about the details of the semi-structured questions in the following paper [48].

#### 2.4.5. Sentiment Analysis

To assess the user feedback more objectively, we conducted sentiment analysis on the text-based responses of the participants. This technique, besides being utilized to evaluate product reviews [67], has been applied to software engineering tasks such as analyzing developers’ emotions in commit messages, among others [68]. The selected tool for sentiment analysis was built on top of the Stanford CoreNLP Natural Language Processing Toolkit [69]. In particular, the sentiment classifier is built on top of a recursive neural network (RNN) deep learning model that is trained on the Stanford Sentiment Treebank (SST), a well-known data set for sentiment analysis. The scale of the classifier distinguished 5 levels of sentiments starting from very negative to very positive. Table 1 describes the levels of sentiments in more detail. Since the CoreNLP toolkit includes a sentiment classifier that evaluates only at the level of sentences, we decided to calculate a weighted average of the sentences comprising the text block as a way to create an aggregate score. In more detail, our approach associated larger weights with the first and last sentence of a participant’s response. Although not all the answers strictly follow the suggested pattern, nonetheless, the majority of them were very close. Furthermore, this pattern is commonly found in product reviews as well, in which the participants’ responses share a resemblance. Subsequently, the set of evaluated responses to the questionnaire per user was aggregated using a linear average as all questions were considered of equal importance. Likewise, the overall sentiment score was calculated as the linear average on the values of the previous step. Again, we considered all the users equally important in determining the score for the training app and training procedure.

Finally, the questionnaire employed in the process of conducting the sentiment analysis is presented in Appendix B. It consisted of 11 questions, to which the participants were requested to respond and share their opinion on the aspects of the training session and the training app as well.

## 3. Results

### 3.1. UEQ+ Results

**Figure 13:**
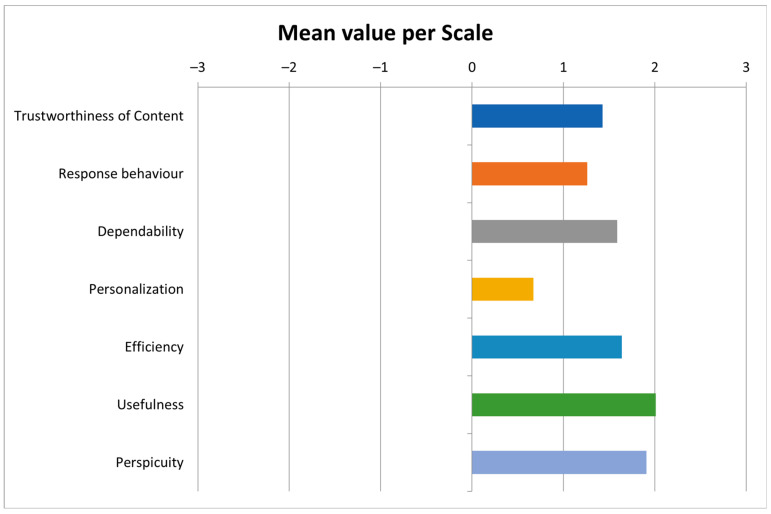
Mean value per scale.

The UX analysis demonstrated that the training application was positively evaluated by the users. In particular, the scale of Personalization, which concerns the customizability of the user’s personal preferences, received the lowest score (Mean = 0.67). The top two scores were assigned to the scales of Usefulness (Mean = 2.01) and Perspicuity (Mean = 1.91) as the users found that the training application both helps them in understanding the functionality of the main application and does it in an easy-to-learn way. Closely following are the scales of Efficiency (Mean = 1.64) and Dependability (Mean = 1.59) as the users considered the training application fast and responsive, and reliable, respectively. Furthermore, the scale of Trustworthiness of Content received a relatively high score (Mean = 1.43) as users consider the provided information of high quality, while the score assigned to the scale of Response Behavior (Mean = 1.26) suggested a desire for better quality characteristics regarding the app’s issued instructions. Finally, the Key Performance Indicator (KPI), an overall assessment metric provided by the UEQ+ statistical tool, received a score of 1.53, which is considered a positive result. Figure 13 below shows the mean values of the scales presented above in a graphical form.

**Figure 14:**
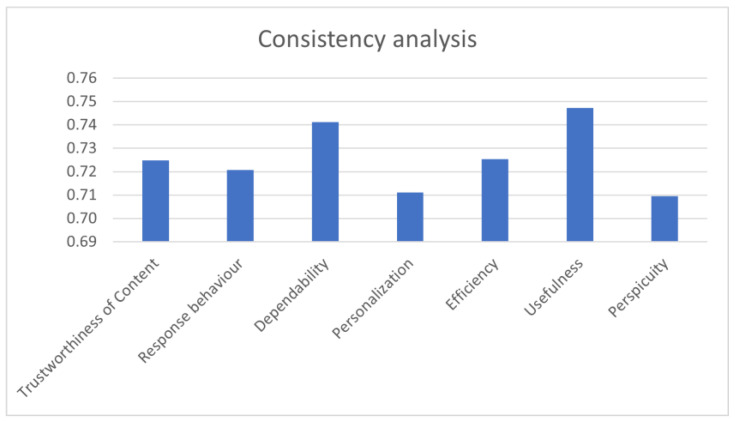
Cronbach’s alpha coefficient.

Cronbach’s alpha coefficient was used to assess the validity of the results for each scale. The results are presented in Figure 14. From there, we can see that each scale passed the threshold value for validity (0.7).

**Figure 15:**
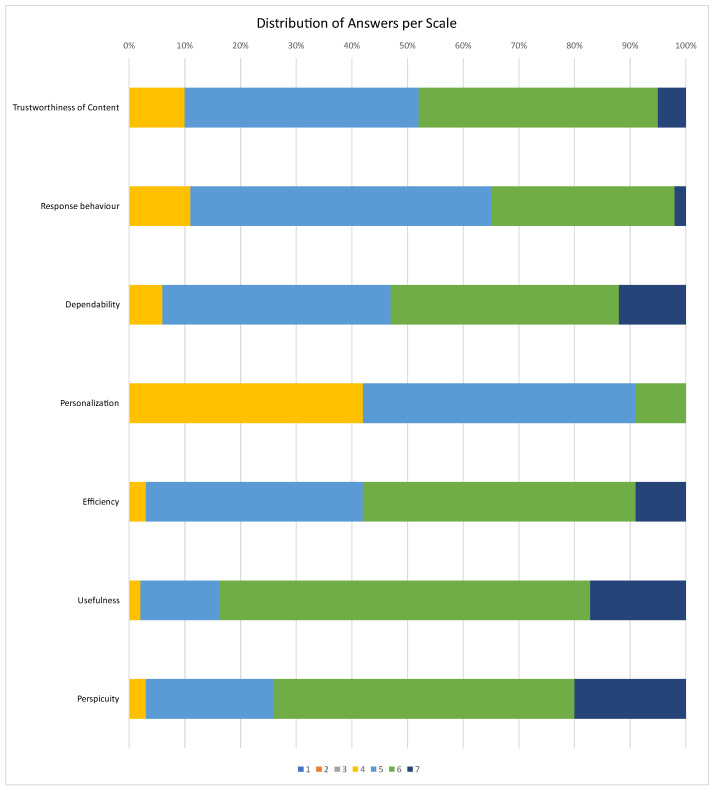
Distribution of answers per scale.

Finally, we conclude the presentation of the UX analysis with a demonstration of the distribution of answers per scale (Figure 15). The totality of the responses ranged from 4 to 7 with the vast majority receiving scores between 5 and 6. The only exception to this is the scale of Personalization which received 4 for a large portion of responses, thus justifying the lower overall score it received.

### 3.2. SUS Results

**Figure 16:**
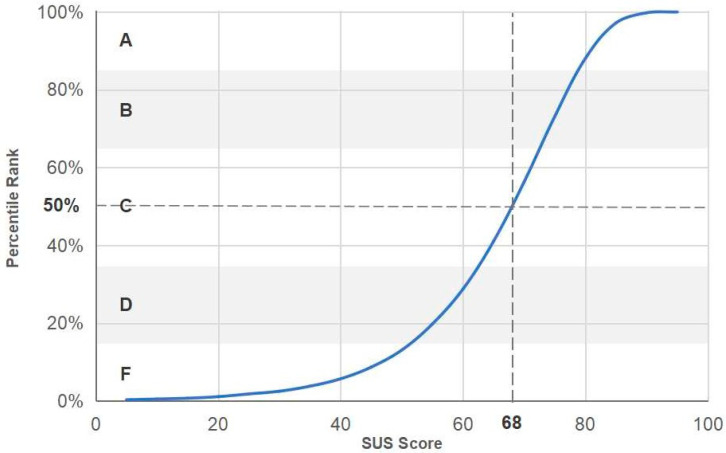
SUS score—Percentiles are assigned grades from A to F.

**Figure 17:**
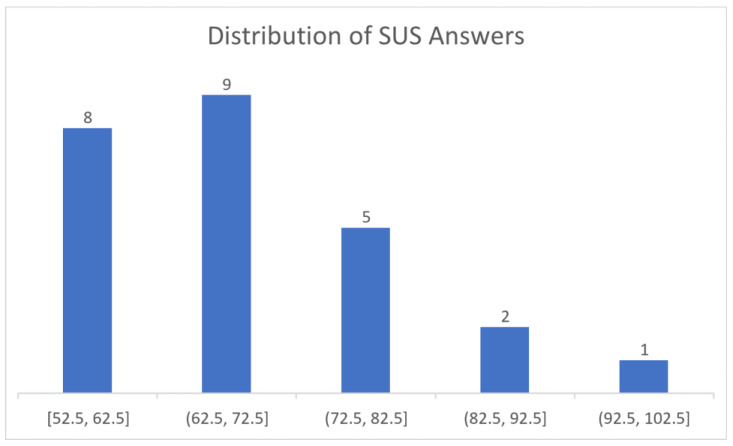
Distribution of SUS Answers.

The overall score for SUS was 69,1, which according to [70] is marginally above the threshold value of 68 (Figure 16). The answers given by the users revealed a balanced view of the training application’s features with an overall positive attitude. Although there is no particular aspect that either stands out or is severely criticized, we could say that the users found the training application’s functionality very well integrated while they found the overall application slightly complex. Furthermore, the evaluation scores ranged between 52.5 and 95. Figure 17 presents the distribution of the scores of the participants. As can be seen, 88% of the participants’ responses fall in the range of 52.5 and 82.5 while more than half of the responses are confined within the ranges of 52.5 and 72.5.

### 3.3. Sentiment Analysis Results

The users’ sentiments were mostly positive with a few neutral assessments; thus, the overall assessment was deemed to be positive. Besides the results of the sentiment analysis, the questionnaire also provided feedback that can give us the opportunity to further improve our approach. Table 2 presents the overall assessment scores per user.

Table 3 presents an example of the answers given by a participant and how the sentiment classifier evaluates them.

## 4. Discussion

Throughout the development of both the main and training application, we overcame a lot of challenges, identified potential areas of improvement, and gained useful insights.

### 4.1. Lessons Learned with the Obstacle Detection System

The obstacle detection system [49], which was developed in the context of the MANTO project [41], was leveraged to train people with blindness and visual impairments by associating its obstacle detection distance capabilities with varying intensity pitch or haptic feedback. In this way distance information was encoded in an accessible format for the visually disabled providing a smartphone-based augmented reality feature. However, due to the currently employed cost-effective wide ultrasonic beam sensor, there are restrictions as far as it concerns the spatial configuration of the training location. From trials with the ultrasonic sensor HC-SR04, we concluded that its characteristics in conjunction with the developed obstacle detection framework are suitable for sparse city environments and, thus, our choice of training spaces was influenced by that fact. In contrast, experimentation with narrow/pencil beam ultrasonic sensors and various configurations of the obstacle detection framework demonstrates the effective application of the solution in dense city environments as well. However, these COTS sensors are considerably more expensive and, thus, might not be feasible to utilize in training sessions where the probability of breaking is high. 

### 4.2. Guidelines Concerning the Training Process and Training App

Throughout our ongoing effort of designing and implementing applications targeting groups of people with special needs, we have found that it is most beneficial when a cognitively informed approach is followed [42]. The latter is an extension of the common iterative process followed in the engineering method with cognitive-based concepts that describe the mental processes utilized during problem-solving. This integration results in solutions that are very close to how individuals address their day-to-day challenges, thus making their adoption easier and, simultaneously, reducing the high abandonment rates. This framework considers, also, as first-class criteria the pillars of safety, reliability, reinforcement, and preferences, as well as incorporating the immediate beneficiaries in the design process. Coupled with the above approach is the requirement to consider the users’ needs in the broader social context as it makes clear the social implications of the available design choices, thus significantly pruning the design space. 

The recommendations given in [71] were found to be effective. In particular, the authors suggest the employment of real-time object detection methods, relatively short in duration training sessions, easy-to-carry and use devices, limited communication of information pertinent to the situation for safety reasons, awareness regarding the social implications of the design choices, as well as the adoption of procedures that ensure the privacy and security of the user’s data. 

Another useful consideration is to recognize that individuals who are blind or visually impaired are equally skilled as sighted individuals with the distinction of being unable to access the wealth of environmental information [43]. Due to the diversity in the amount and kind of environmental information individuals acquire and store, adaptable solutions are required to prioritize differentiated needs and preferences.

Crucial to the design of effective training applications is the input given by the O&M specialists as well. These specialists have invaluable information on what works regarding the processes and techniques employed to effectively teach orientation and mobility skills to the blind and visually impaired. Moreover, from our experience, the O&M courses can be leveraged to introduce new applications and features as individuals with blindness and visual impairments are more receptive since it takes place in locations familiar to them. Working in small groups preferably brings better results, and organizing the available functionality to enable and promote self-discovery is important.

From a technological point of view, location-aware mobile devices can play an important role in spatial learning by detecting current user context and location, utilizing logging and, subsequently, analyzing navigational path traces to determine the user’s routines.

Controlling the amount of the given information is critical to the success of the training application. Participants expressed their requirement to be in control, even with a push-based style of interaction, to determine when and the amount of information they would like to receive. Furthermore, as a better form of control the participants requested a combination of push and pull style of interaction, where they request information from the system and, in turn, the system continues to provide information to them for a period of time before fading out.

Given users prefer the combination of push- and pull-based styles of interaction, as mentioned above, the next step is to find the correct balance for the training application. In contrast to the main application where the interaction should be geared more in favor of push-based interaction since the main responsibility is to transmit navigational instructions and other relative information, the training application instead needs to provide the users with the necessary options to control at their own will the required information in order to facilitate the learning process. 

Finally, another suggestion made by the participants concerned the use of audio cues instead of issuing navigational instructions to signify the types of places and other points of interest they are passing by, and thus expedite the process of tracing the virtual path. 

### 4.3. Virtual Reality, Training, Adoption and Overcoming Challenges

Over the last 25 years, researchers and developers have worked on developing multisensory VR-based systems to help blind individuals develop orientation skills. The advantages of these solutions as highlighted in previous works include improved spatial information perception, solving spatial problems, practicing and improving O&M skills, and developing O&M strategies [24,26,28], as well as enabling the user’s independent interaction, displaying immediate feedback tailored to the user’s sensory and cognitive abilities, and providing the opportunity to practice in a safe environment without time or professional constraints. Furthermore, virtual environments (VEs) can facilitate the work of O&M professionals in providing better training services [33]. Most VR systems contain both indoor and outdoor areas, allowing blind learners to preview a new environment ahead of time. In this way the learner while exploring the virtual environment can interact with landmarks and clues, thus collecting spatial information critical for the construction of cognitive maps applicable to real environments.

Smartphone-based AT applications, a complementary solution that offers virtual environment immersion with accessibility to a massively larger population, play an important role in helping blind people conduct their life with as much independence as possible. Training them with regard to the usage of these applications is important to increase the chances they will keep using them in the future,. However, a number of other issues hindering the adoption of smartphone-based ATs need to be addressed as well. Specifically, these can be the result of either environmental conditions or specific design choices. 

One of the environmental challenges affecting the adoption of smartphone devices is the case of situational impairments, as they have been shown to degrade the performance of users. The study by [72], identified with the help of various participants several such factors that negatively affected their ability to use their smartphone devices. Specifically, using the smartphone device while walking presented challenges to some participants as it reduced motor control over situational awareness and made it impossible to listen to sounds in the environment. Further compounding the challenge of using smartphone devices while walking is the case that when other tasks are involved, situational awareness can be degraded even more. This is backed up by previous research that shows performance degradation from using a smartphone device during these kinds of circumstances, demonstrating simultaneously that these effects may be more adverse for people with visual impairments. This suggests that it may not be possible to use smartphone devices without reducing situational awareness [73].

### 4.4. Design Decisions and Challenges

The challenges related to the design decisions made for smartphone-based applications include the following: (1) gestures-related issues; (2) a lack of consistency in the applications as there is no single way to access a feature; (3) different interfaces per application leading to confusion; (4) non-accessible-friendly features for non-visual users; and (5) issues related to learning to use the talkback service by novices. This list is by no means exhaustive.

In order to address the challenges and deficiencies despite the selected technological approach, resources are required to aid the adoption process. However, our research team discovered that there is a scarcity of those relevant resources available, further compounding a difficult problem that is both time-consuming and difficult to undertake. Currently, it is expected that the users be persistent and willing to ask for aid. Especially the latter is impossible to eliminate no matter how well-designed a solution may be, as has been demonstrated by all of these years of research. Furthermore, even with the progress made where many challenges have been identified, there are several still overlooked or underexplored [74]. Below we provide a comprehensive list of open challenges that future research needs to address to achieve better smartphone-based accessibility:

- Learning and exploring—Challenges related to learning and performing movements on touchscreens have not yet been overcome, despite the effort put into that area. It remains difficult for individuals with blindness and visual impairments to discover and learn based on any given description, leaving them only with their support network for substantive assistance.
- Adapting mental models—New releases of the widely available operating systems and applications usually bring new changes to the existing interfaces, without any accompanying relevant descriptions in an accessible format, thus forcing users to adapt their daily routines to the new conditions every time a redesign of user experience occurs.
- Accessibility of applications—Although there is a great number of efforts targeting accessibility aspects of smartphone applications, the results are fragmented without providing a common frame of reference or any sort of actionable advice.
- Forced interfaces—The choice of a touchscreen interface does not seem to be the most appropriate one for blind users. Instead, a redesign of smartphones for the target group having more physical buttons could be a step in the right direction.
- Ubiquitous accessibility information—Individuals with blindness and visual impairments require access to a centrally available repository of information relative to accessibility issues for applications and devices, to facilitate the adaptation of the users’ mental model caused by the ongoing non-standard interface changes introduced in each re-iteration. Users might be able to make meaningful choices with the help of a dedicated accessibility rating and other statistics.
- Enabling sharing and peer support—Many individuals find no support for their cases as it is either inaccessible or incompatible with their device configuration. Rodriguez et al. in [75] identified the shortcomings of the current communication methods that include asking questions of other people and/or searching online as both being time-consuming and removing the user from the context of the problem often providing no results. To address and achieve effective communication in an accessible manner, the right understanding and tools are required.

Finally, our prioritization for the future concerns the fine-tuning of the existing version of the simulation app as well as the design, implementation, and validation of a VR application that will hopefully improve the trainability of the blind and visually impaired. 

## 5. Conclusions

We created a supplemental training version, functionally equivalent to the main application, to help the user become familiar with the provided features in the context of training courses. This was a highly demanded request as besides learning to use the application itself, the training sessions can be used for acquiring long-term Orientation and Mobility skills. Furthermore, this demand is provoked partially by the varying skills blind individuals have in using complex technologies as well as from the challenges arising from the interaction with a complicated and dynamic environment. 

The way forward in overcoming these challenges and somewhat reducing the burden on the blind and visually impaired is the provision of simulation-based navigation applications that incorporate in their process the use of familiar equipment and, at the same time, enable users to repeatedly navigate routes at their own pace and location. Additionally, another benefit of a simulation application is its friendliness and flexibility in not having to carry special equipment.

In our effort to design an effective training tool, we searched the literature for any shortcomings in the process related to learning about route navigation in complex environments. Typically, various in-situ navigation tools, tactile maps, and virtual navigation solutions are used to facilitate the previously mentioned process. Nonetheless, these tools are time-consuming whereas our application avoids this issue by being less restrictive. Another added benefit of our application is the capability to protect the user from real-life hazards while providing a very close-to-reality simulation of the navigation process.

Users were, also, asked to review their interactions with the application and the educational process as soon as the training sessions were over.

In general, most of the users evaluated the above process positively. Sentiment analysis on user responses confirmed the Usability and UX results. Finally, we concluded with the lessons learned and designated open challenges and future directions for achieving better smartphone-based accessibility.

## Figures and Tables

**Table 1:** Sentiment Analysis Levels.

Level	Sentiment
0	Very Negative
1	Negative
2	Neutral
3	Positive
4	Very Positive

**Table 2:** Assessment scores per user.

Participant	Sentiment	Participant	Sentiment	Participant	Sentiment
#1	3	#2	3	#3	3
#4	3	#5	3	#6	3
#7	3	#8	3	#9	3
#10	3	#11	3	#12	2
#13	3	#14	3	#15	3
#16	3	#17	3	#18	3
#19	3	#20	3	#21	3
#22	3	#23	3		

**Table 3:** Sample of sentiment classifier mappings from answers to scores.

Answer from Participant #2	Evaluation
“It was so-so. I wanted the training application to have more routes available”	2
“The instructions made me feel at ease. I am very happy.”	3
“Yes, it was very convenient.”	2
“Yes, I prefer the orthogonal instructions.”	2
“I would prefer it to be better.”	2
“It is not representative of the real case.”	1
“Yes. It makes me feel confident as to what I have to do when entering and exiting a bus.”	3
“Yes. I think it does a good job of describing the situation. “	3
“The screen read worked fine. “	3
“Yes. It was easy for me.”	3
“I think it gives a good idea as to what to expect from the main application.”	3

## Data Availability

Not applicable.

## Appendix A

**Table A1:** SUS Questionnaire.

# 1	Item
SUS-1	I think that I would like to use this system frequently.
SUS-2	I found this app unnecessarily complex.
SUS-3	I thought the system was easy to use.
SUS-4	I think that I would need the support of a technical person to be able to use this app.
SUS-5	I found the various functions in this app were well integrated.
SUS-6	I thought there was too much inconsistency among the app functionalities.
SUS-7	I imagine that most people would learn to use this app very quickly.
SUS-8	I found the app very cumbersome to use.
SUS-9	I think I would feel very confident using this app.
SUS-10	I needed to learn a lot of things before I could get going with this app.

## Appendix B

This questionnaire was used to receive feedback from the users on the training procedure as well as to utilize the collected data to conduct sentiment analysis to reach automatically a conclusion concerning the attitude of the users.

Training course assessment questionnaire:

1. Was the training application helpful in becoming accustomed to the main application’s functionality?
2. Are the command instructions accurate and helpful enough to control the application?
3. Is minimal action attainable for completing tasks? Does the completion of tasks require a minimal number of actions? (Minimal action)
4. Are the available formats of the issued instructions (orthogonal or clockwise) satisfactory enough?
5. Is the example concerning the recovery from an error back to the correct navigational path satisfactory?
6. Is the scenario concerning the obstacle detection device illuminating?
7. Is the scenario concerning the combined navigation with public means of transportation illuminating?
8. Is the scenario concerning passing traffic light crossing illuminating?
9. Is the training application compatible with screen readers?
10. Is the training procedure easy?
11. Would you consider the training application attractive to you?
